# Effect of simulated warming on leaf functional traits of urban greening plants

**DOI:** 10.1186/s12870-020-02359-7

**Published:** 2020-04-03

**Authors:** Jiyou Zhu, Hua Zhu, Yujuan Cao, Jinhang Li, Qiuyu Zhu, Jiangming Yao, Chengyang Xu

**Affiliations:** 1 Key Laboratory for Silviculture and Conservation of Ministry of Education, Key Laboratory for Silviculture and Forest Ecosystem of State Forestry Administration, Research Center for Urban Forestry, Beijing Forestry University, Beijing, 100083 China; 2 Inspection Department of Guangxi Medical College, Nanning, 530402 China; 3 Guangxi University, Nanning, 530005 China

**Keywords:** leaf functional traits, Leaf economics spectrum, Warming, Urban greening, Adaptation strategy

## Abstract

**Background:**

Response and adaptation strategies of plants to the environment have always been the core issues in ecological research. So far, relatively little study exists on its functional traits responses to warming, especially in an urban environment. This information is the key to help understand plant responses and trade-off strategy to urban warming.

**Results:**

We chose the common greening trees of mature age in Beijing (*Fraxinus pennsylvanica, Koelreuteria paniculata, and Sophora japonica*) as the research subjects, and used infrared heaters to simulate warming for three gradients of natural temperature (CK), moderate warming (T1) and severe warming (T2). Results showed that:(1) Leaf dry matter content (LDMC), chlorophyll content (CHL), leaf tissue density (LTD), and stomatal density (SD) all increased with temperature warming. Specific leaf area (SLA), stomatal size (SS), and stomatal aperture (SA) decreased with simulated warming. (2) SLA was extremely significantly negatively correlated with CHL, LDMC, LTD and SD (*P* < 0.01), and was extremely significantly positively correlated with SS (*P* < 0.01). SA was extremely negatively correlated with SD (*P* < 0.01), and was extremely significantly positively correlated with SS (*P* < 0.01). There was a significant positive correlation between LDMC and LTD (*P* < 0.01). This showed that urban greening trees adapted to the environment by coordinating adjustment among leaf functional traits. (3) Under the T1 treatment, the *R*^2^ and slope among the leaf traits were higher than CK, and the significance was also enhanced. The correlation between leaf traits was strengthened in this warming environment. Conversely, it will weaken the correlation between leaf traits under the T2 treatment.

**Conclusion:**

Our study demonstrated that there was a strong trade-off between leaf functional traits in the urban warming environment. Plants in the warming environment have adopted relatively consistent trade-offs and adaptation strategies. Moderate warming was more conducive to strengthening their trade-off potential. It is further verified that the global leaf economics spectrum also exists in urban ecosystems, which is generally tend to a quick-investment return type with the characteristics of thick leaves, strong photosynthetic capacity, low transpiration efficiency and long life in urban environments.

## Background

Global climate warming has become an indisputable fact [1, 2]. Previous study pointed out that the global average temperature has continued to rise at an unprecedented rate in the last four decades, and the global mean temperature is likely to increase by 1.1–6.0 °C in the next century [3], especially in areas where the urbanization is accelerating [2, 4, 5]. Under the background of global warming, the intensity of urban heat islands has also increased [6]. This has changed the growth environment of urban greening plants with the continuous advancement of urbanization [6, 7]. Global warming directly leads to an increase in atmospheric temperature and surface temperature, which will affect the survival, growth, development, and reproduction of urban vegetation [8]. Some are likely to adapt to the warmer temperatures environment by changing and adjusting their phenotype or physiological structure [8, 9]. However, due to their own growth characteristics, some species may not be able to adapt to high-temperature environments and grow poorly or even diet [10]. Research on the leaf functional traits and the leaf economics spectrum in urban plants after warming is still insufficient so far. Furthermore, researchers pay great attention to the growth of mature trees in urban forestry research [11]. However, most of the methods of current research are mainly greenhouse control experiments targeting young seedlings, which cannot objectively reflect the characteristics of the urban environment [12, 13]. In addition, previous studies focused on the response of seedling growth to warming, whereas lack of research on the response of mature trees to temperature changes [13, 14]. Due to the different growth stages and developmental conditions, the adaptation strategies of saplings to the environment are not fully applicable to mature trees. Therefore, it is necessary to study the response of urban greening plants to warming.

In the long-term adaptation of the living environment, plants gradually form a series of morphological and physiological structural traits that are interrelated, co-evolving and adapt to changes in the external environment, collectively referred to as plant functional traits [15, 16]. Moreover, plants often respond to environmental changes by adjusting their functional traits [17, 18]. In recent years, studies based on the relationship between leaf functional traits and environmental factors have shown that leaf functional traits can better reveal plant survival, growth and adaptation strategies, and ultimately expressed in the organization and physiological characteristics of plant organs [19–21]. The leaf economics spectrum refers to a series of regular and continuously changing combinations of plant leaf functional traits [22]. Since the concept of the global plant leaf economics spectrum was put forward, many researchers have carried out exploratory research in different spatiotemporal scales and different ecosystem types, which verified the widespread existence of LES [22, 23]. However, relevant researches in China are mostly limited to theoretical basic researches [21–24]. Therefore, exploring the growth, survival and development process of plants, especially the response of plant functional traits and their leaf economics spectrum (LES) to simulated temperature rise, is conducive to predicting the impact of climate warming on plants [25, 26].

Although some studies have carried out a series of explorations on the effects of plant functional traits under different environmental conditions, most of them were confined to forest ecosystems in low mountains or alpine regions [27]. In addition, few studies explained the adaptation of greening plants in urban environments to urban high temperatures environment. It is not clear whether the global leaf economics spectrum exists in the urban environment so far, and whether urban greening plants will show some trade-off strategy in order to adapt to the changing environment. In view of this, our study attempted to use the typical greening plants in Beijing as the research object, simulate the warming by infrared radiant heaters. And we observed the changes of plant functional traits in an urban environment, aiming to explore the response of plant functional traits to warming and its trade-off rules, and verifying the existence of plant economics spectrum. At the same time, the important index of stomatal characteristics is included in the research scope, further enriching the study of the global leaf economics spectrum, including its research area, object, and indicator parameters [28, 29]. Based on the sensitivity of leaf functional traits to changes in the external environment, we suspect that plants may also adjust their leaf functional traits to adapt to environmental changes under conditions of increasing urban temperatures. It will help to clarify the response mechanism of urban greening plants to short-term warming and provide a theoretical reference for global warming research.

## Results

### Relationship between functional traits of leaves and their response to warming

#### Effect of warming on plant leaf functional traits

**Fig. 1:**
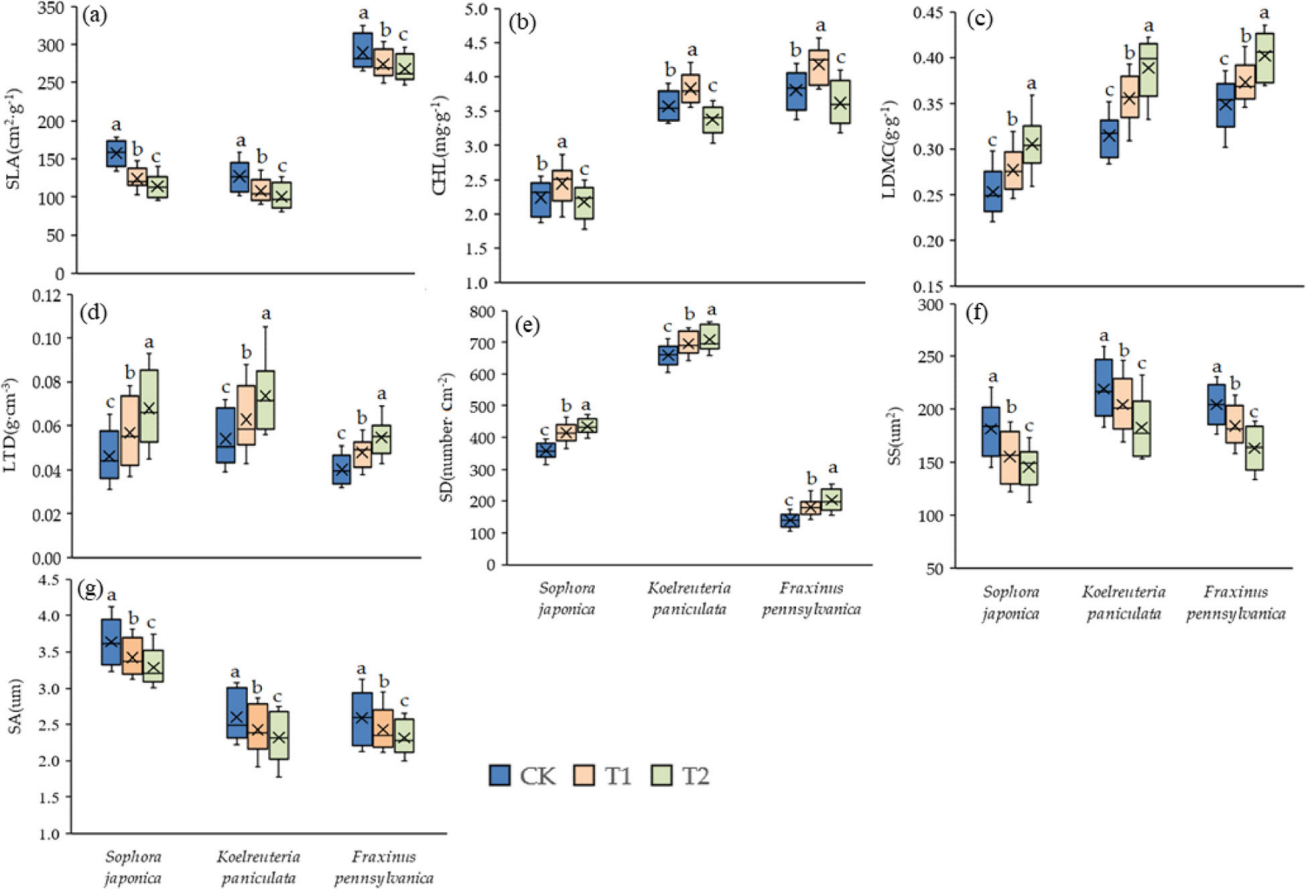
Effect of different warming treatments on plant functional traits. Different lowercase letters indicate that the indicator difference reaches a significant level. **a**: Specific leaf area, **b**: Chlorophyll content, **c**: Leaf dry matter content, **d**: Leaf tissue density, **e**: Stomatal density, **f**: Stomatal size, **g**: Stomatal aperture

The specific leaf area, stomatal aperture and stomatal size of the greening trees were continuously reduced under the warming treatment. Among the three temperature regimes, T1 and T2 treatment significantly reduced the specific leaf area, chlorophyll content and stomatal size of greening trees (*P* < 0.05). Compared with CK, T1 and T2 significantly increased leaf dry matter content, leaf tissue density, stomatal density and stomatal aperture of three greening plants, and T2 was more significant (*P* < 0.05). Compared with CK and T2, T1 significantly increased chlorophyll content (*P* < 0.05), indicating that small-scale warming was beneficial to increase plant chlorophyll content (Fig. 1). It is worth noting that the leaf traits of the three common urban greening species were consistent under different warming treatments (CK, T1 and T2).

#### Correlation between leaf functional traits

As shown in Table 1, there was a significant correlation between the functional traits of greening plant leaves under the urban thermal environment effect. Among them, there was an extremely significant negative correlation between specific leaf area and chlorophyll content, leaf dry matter content, leaf tissue density, stomatal density (*P* < 0.01), but showed an extremely significant positive correlation with stomatal size (*P* < 0.01). There was an extremely significant positive correlation between leaf dry matter content and leaf tissue density (*P* < 0.01). There was a negatively significant negative correlation between stomatal density and stomatal aperture (*P* < 0.01), but stomatal aperture and stomatal size showed an extremely significant positive correlation (*P* < 0.01). The functional traits of three common greening plants showed a strong and weak regulation relationship. This indicated that the leaf functional traits showed a certain trade-off strategy in order to adapt to the urban environment, which verified the existence of the leaf economics spectrum in the urban forest ecosystem.

**Table 1:** Pearson correlation analyses of plant functional traits in different warming treatments. ** indicates that the traits are extremely significant at the level of *P* < 0.01. * indicates that the traits are significantly different in the level of *P* < 0.05

	Warming treatments	CHL	SLA	LDMC	LTD	SD	SS	SA
CHL	CK							
	T1							
	T2							
SLA	CK	−0.538**						
	T1	−0.721**						
	T2	−0.586**						
LDMC	CK	0.187	−0.480**					
	T1	0.223	−0.645**					
	T2	0.192	−0.580**					
LTD	CK	0.102	−0.526**	0.599**				
	T1	0.137	−0.621**	0.708**				
	T2	0.100	−0.570**	0.520**				
SD	CK	−0.159	−0.604**	0.104	0.245			
	T1	−0.222	− 0.713**	0.110	0.267*			
	T2	−0.134	−0.661**	0.098	0.232			
SS	CK	0.240	0.607**	−0.127	−0.218	−0.153		
	T1	0.289*	0.617**	−0.213	−0.258*	− 0.246		
	T2	0.229	0.463**	−0.213	−0.217	− 0.169		
SA	CK	0.239	0.207	−0.142	0.128	−0.380**	0.361**	
	T1	0.287*	0.251	−0.159	0.244	−0.400**	0.478**	
	T2	0.190	0.190	−0.153	0.136	−0.363**	0.469**	

#### Effect of warming on the relationship between plant leaf functional traits

**Fig. 2:**
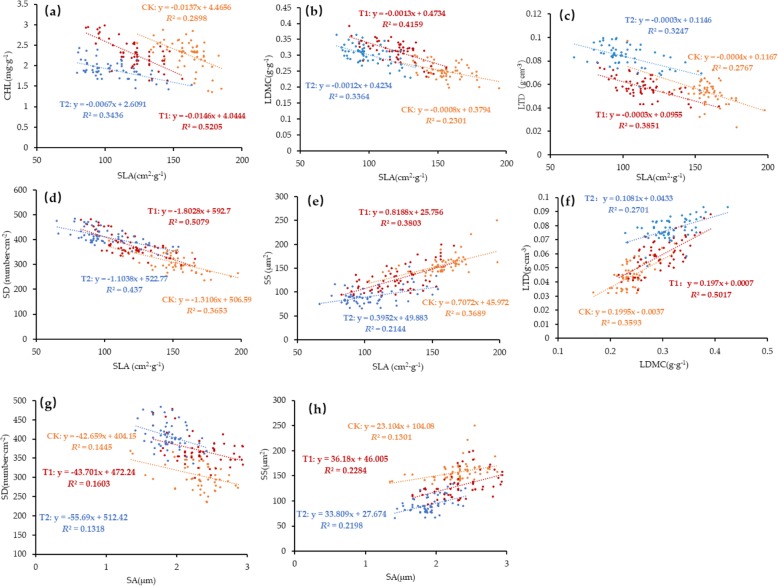
Effect of warming on the relationship between plant functional characters. **a**: SLA and CHL correlation, **b**: SLA and LDMC correlation, **c**: SLA and LTD correlation, **d**: SLA and SD correlation, **e**: SLA and SS correlation, **f**: LDMC and LTD correlation, **g**: SA and SD correlation, **h**: SA and SS correlation

**Fig. 3:**
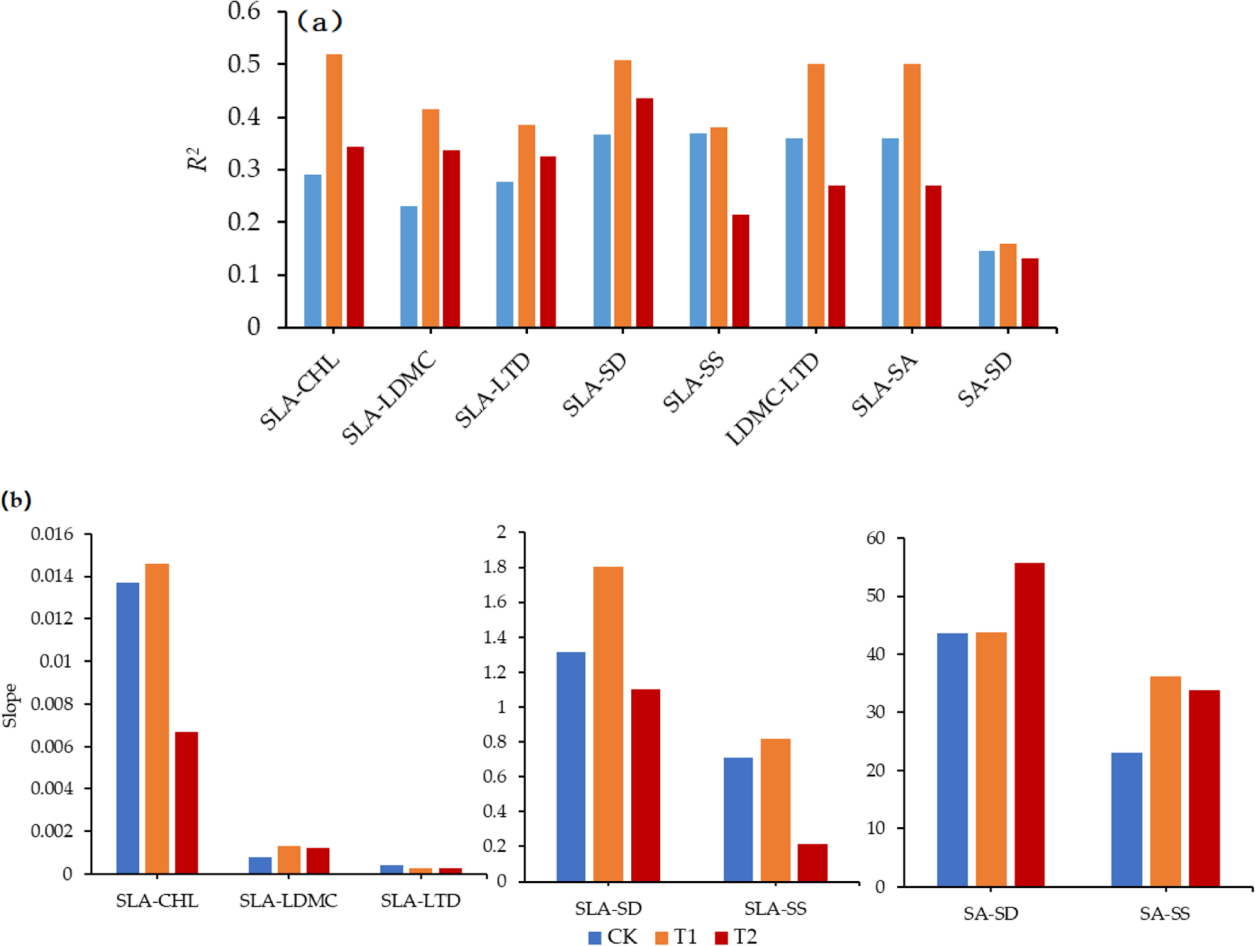
*R*^2^ and slope among leaf functional traits in different warming treatments. **a**: *R*^2^, **b**: slope

As shown in Table 1, the correlation between functional traits of plant leaves showed a trend of increasing first and then decreasing under different warming treatments. Compared with CK, the Pearson correlation coefficient between functional traits increased at T1 and decreased at T2. As shown in Fig. 1 and Fig. 2, there was a significant correlation between leaf functional traits in different warming treatments, but there were some differences in their correlation degree. As shown in Table 2, different warming treatments have different effects on the regression slope. Compared with CK, the regression slopes of chlorophyll content & specific leaf area, specific leaf area & stomatal size were all significantly higher than T2 (*P* < 0.05). In the warming treatments (T1 and T2), the regression slope differences between specific leaf area & stomatal density, specific leaf area & stomatal area, leaf dry matter content & leaf tissue density reached significant levels (*P* < 0.05). In the linear regression analysis between functional traits, the value of *R*^2^ and slope increased first and then decreased with the increase of warming degree. Under the small temperature increase treatment (T1), the value of *R*^2^ and the value of slope between the leaf functional traits were higher than that of CK, and the saliency was also enhanced. On the contrary, when the temperature further increases (T2), the value of *R*^2^ and the value of the slope between leaf functional traits showed a downward trend in general (Fig. 3). According to the correlation of *R*^2^, slope and Pearson correlation, this fully demonstrated that the small increase in temperature enhanced the correlation between leaf functional traits, and the large temperature increase of T2 weaken the correlation between plant leaf functional traits.

**Table 2:** Significant test of the regression slope difference between plant functional traits under different warming treatments based on univariate linear model analysis. * indicates that the traits are significantly different in the level of *P* < 0.05

	CHL-SLA	SLA-LDMC	SLA-LTD	SLA-SD	SLA-SS	LDMC-LTD	SLA-SA	SA-SD
CK-T1	0.783	0.086	0.481	0.134	0.554	0.957	0.813	0.956
T1-T2	0.607	0.671	0.735	0.014*	0.014*	0.013*	0.846	0.607
CK-T2	0.017*	0.213	0.337	0.457	0.000*	0.031	0.942	0.578

## Discussion

Cities as a comprehensive system of society, economy, and ecology, are very important places for human activities and life [30]. Urban greening plants are more sensitive to changes in climatic conditions in such a mixed environment [31, 32]. With the advancement of urbanization and the gradual warming of the global climate, changes in the urban thermal environment have also had a series of effects on urban greening plant growth [33]. Previous studies have reported that plants undergo functional and morphological regulation and adjustment in response to changes in the living environment during long-term evolution, growth, and development [17, 19, 20]. In our research, we further verified that the functional traits of urban greening plants have made a trade-off strategy in the urban warming environment.

### The response of urban greening plant functional traits to warming

Increasing the temperature promotes the reduction of the specific leaf area of ​​the plant. Previous studies have reported that specific leaf area can accurately represent the adaptability of plants to the environment, the acquisition of resources and the ability of self-protection in strong light. It is closely related to drought resistance, high-temperature resistance and photosynthetic capacity of plants [33–35]. Many studies have pointed out that the low specific leaf area uses most of the resources for the construction of self-defense structures to resist the interference of adverse environment. However, a high specific leaf area usually has higher productivity and is suitable for living in habitats with sufficient nutrients [36]. This study found that urban greening plants tend to exhibit high temperature and drought tolerance characteristics in functional traits. Three typical greening plants, such as *Fraxinus pennsylvanica, Koelreuteria paniculata,* and *Sophora japonica*, were significantly lower than leaf area in the warming environment. The results showed that the increase of temperature promoted the utilization of resources, materials and energy obtained by most greening plants, thus forming small and thick leaves and extended the moisture inside the leaves to the outside. This strategy may be to avoid damage to plants caused by high temperature and prevent plants from dying due to excessive water loss, which is basically consistent with the findings of many scholars [37–39]. Experimental warming promoted the increase of chlorophyll content in the leaves of plants. Most of the energy for photosynthesis in plants comes from the light energy captured by photosynthetic pigments, so the level of chlorophyll content is closely related to the photosynthetic capacity of plants [40, 41]. Studies have shown that warming enhances the photosynthetic capacity of plants by accelerating the synthesis of photosynthetic pigments in plant leaves, thereby promoting their growth and development [42, 43]. The increase of chlorophyll content is a suitable way for plants to cope with the increase of ambient temperature or light enhancement, so as to facilitate their full photosynthetic conditions under the condition of limited leaf area [44–46]. Our study found that small-scale warming could significantly increase the chlorophyll content of *Fraxinus pennsylvanica, Koelreuteria paniculata* and *Sophora japonica*, which indicated that small-scale warming treatment could provide a better temperature environment for the synthesis of plant photosynthetic pigments, thereby promoting plant photosynthetic capacity. Conversely, the chlorophyll content of plants under severe warming treatment decreased, indicating that heavy warming may inhibit the synthesis of photosynthetic pigments. Plants can complete the normal operation of photosynthesis within a certain temperature range, and measures to reduce the chlorophyll content in the excessively high-temperature environment to reduce the absorption of light energy by the leaves to avoid high-temperature burns.

Experimental warming significantly increased the leaves dry matter content. The leaves dry matter content mainly indicates the ability of plants to retain nutrients and has a good indication of the amount of input into the blade construction [47, 48]. Previous studies have shown that the density of plants will increase in an environment rich in nutrient elements, which will increase the competitive pressure of plants on resource utilization. Under such conditions, this will lead to a significant reduction in the plant’s ability to retain nutrients, which is manifested by a reduction in the leaves dry matter content in terms of leaf functional traits [41, 47]. The results of this study showed that the warming treatment promoted the increase of dry matter content in plant leaves. Moreover, the increase of leaf dry matter content gradually increases with the increase of ambient temperature. This indicated that plants maintain a more stable nutrient retention capacity in an environment with elevated temperature, which was conducive to adapt to high-temperature stress environment and maintain normal growth. Therefore, the experimental results obtained by us can provide an important reference for the selection and management of urban greening plants, especially the evaluation of high-temperature tolerance of tree species under the background of urban environmental temperature rise.

Experimental warming significantly increased leaf tissue density. The increase of leaf tissue density is beneficial to reduce transpiration, thereby alleviating the water dispersion of plants. At the same time, the increase of leaf tissue density can slow down the growth rate of plants and reserve more carbon for the construction of tissue to adapt to the high-temperature environment of the city [49]. Leaf tissue density reflects the defense ability of leaf tissue, so plants use more substances in the high-temperature environment to increase their leaf tissue density, which is beneficial to plants to make better use of limited resources to adapt to the growing environment. In this study, the leaf tissue density of three common greening tree species was increasing with the increasing of the warming range within a certain temperature range. This showed that urban greening plants put more nutrients to the construction of defense structures to prevent excessive water loss or high-temperature damage under the environment of elevated temperature.

Experimental warming increased stomatal density and reduced the stomatal size and stomatal aperture. Plant stomata, as an important channel for gas exchange, connect plants and the external environment and play an important role in regulating the plant water balance [50]. In urban environments, changes in plant stomatal density are not only related to their own growth characteristics, but also susceptible to environmental factors. Hydrothermal conditions are the main limiting factor for the stomatal density of plant leaves [50–52]. Plants respond quickly to environmental changes through the aperture and closing of stomata, which is the result of the long-term adaptation of plants to the external environment during evolution. Stomatal density and stomatal size are two important parameters that characterize stomatal morphological characteristics. In this study, we found a stable negative correlation between them. Under the temperature-increasing treatment, the stomatal density of plant leaves increased significantly with the decrease of specific leaf area, which promoted the transpiration of plant water. Therefore, the plant adopts a strategy of reducing the stomatal size and the stomatal aperture degree to reduce the loss of moisture in order to adapt to the high-temperature environment.

### Relationship between functional traits of plant leaves and the effects of warming and leaf economics spectrum analysis

**Fig. 4:**
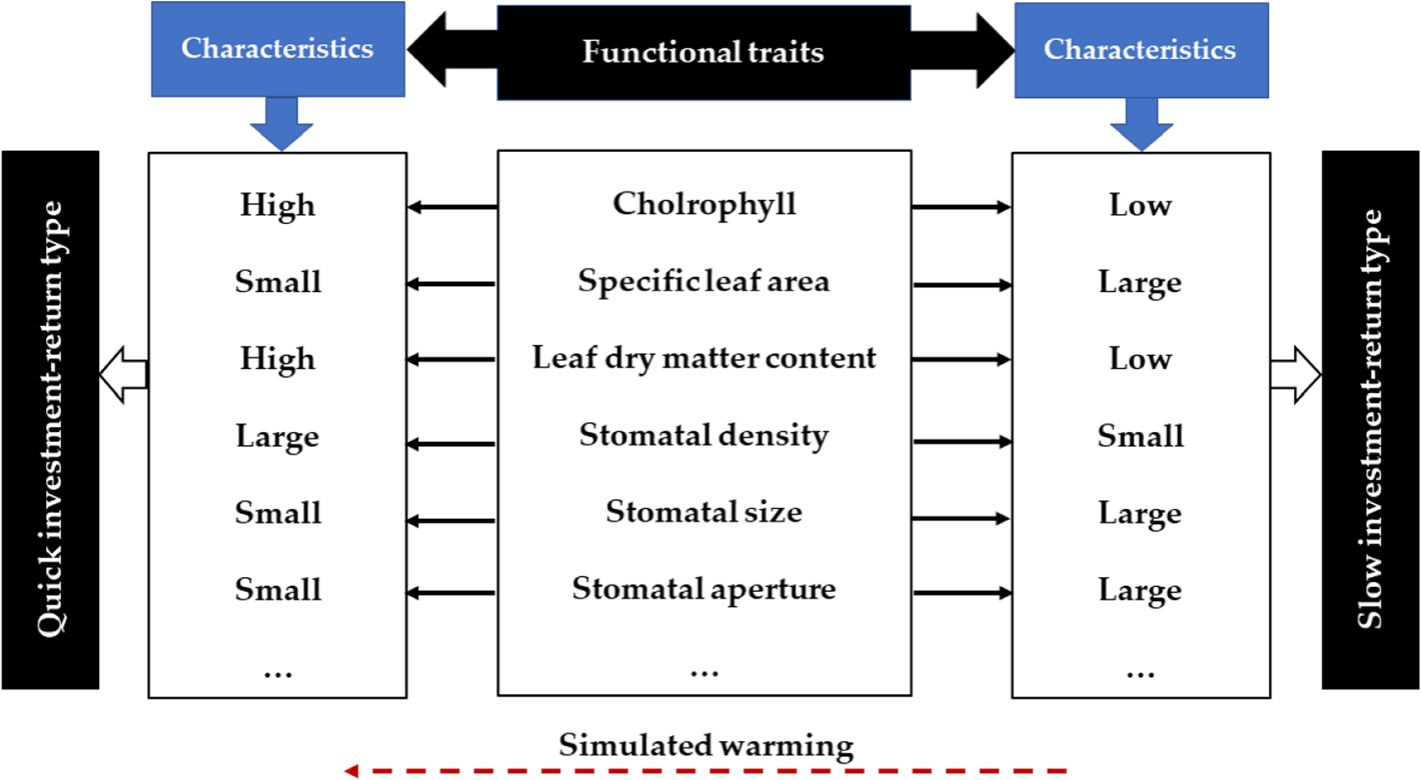
Conceptual illustration of leaf economics spectrum (developed based on Wright et al., 2004). The leaf economics spectrum has two endpoints: one is of “Quick investment-return type” with the characteristics of high chlorophyll, small specific leaf area, high leaf dry matter content, etc. While the other end is of “Slow investment-return type” with the characteristics of low chlorophyll, large specific leaf area, low leaf dry matter content, etc.

This study found that there was a significant correlation between the functional traits of greening plant leaves in urban ecosystems. Among them, there was a significant negative correlation between specific leaf area and chlorophyll content, leaf dry matter content, leaf tissue density, and stomatal density. However, there was a significant positive correlation between specific leaf area and stomatal area. Leaf tissue density and leaf dry matter content showed a very significant positive correlation. This is basically consistent with the findings of many scholars on a global scale and in special habitats [46]. In this study, we added stomatal traits to the research category. We found that stomatal density was extremely negatively correlated with stomatal aperture and was extremely significantly positively correlated with stomatal size. This is similar to the analysis results of the difference between urban heat island and urban cold island on urban greening traits in our previous research [53], further verifying the trade-off between stomatal traits. We found that the leaves of plants became thicker, the leaf area became smaller, the photosynthesis ability was stronger, and their ability to retain nutrients became stronger in the special environment of high temperature. This was consistent with the existing research results [16, 19]. These traits together form the global leaf economics spectrum, reflecting the different ecological strategies that plants use to balance the cost and effort of leaf construction costs. Our current results suggest that leaf functional traits of three common greening plant showed relatively consistent trade-offs and adaptation strategies, further verifying that the global leaf economics spectrum also exists in the special environment of the city, and was biased towards quick investment-return type in the global leaf economics spectrum (Fig. 4). Therefore, under the conditions of climate warming, urban greening plants mainly through the distribution and regulation of resources between traits, thereby accelerating the growth rate and shortening the breeding period to adapt to the warming environment.

There were some differences in the response of different leaf functional traits to experimental warming. In CK and moderate warming treatment, there was a significant correlation among the traits of the leaves, and the large temperature increase weakened the correlation between the functional traits of the leaves. At the same time, under the small-scale warming treatment, the *R*^2^ between the traits of the leaves was significantly higher than that of CK, and the saliency was also enhanced, which indicated that the small-scale warming enhances the correlation between leaf traits. On the one hand, this may be due to the temperature of the small-scale warming range that was still at the optimum temperature range for plant growth. In most cases, the temperature at which plants undergo normal photosynthesis is in the range of 10–35 °C, and the photosynthesis rate generally increases with increasing temperature in this temperature range [14, 54, 55]. On the other hand, we suspected that the correlation between plant traits was related to its resource investment and return strategies under different warming conditions [17, 18, 38–40, 55], and such strategies are generally consistent among different tree species (Fig.1). On the whole, the correlation among traits showed an increasing trend when the temperature was moderate(T1), but a decreasing trend when the temperature was too high (T2). As can be seen from Fig. 1, the plant character values have increasing or decreasing changes under different warming treatments. For example, the specific leaf area decreases gradually with the increase of temperature, while the leaf dry matter content increases with the increase of temperature. As can be seen from Fig. 2, the specific leaf area has a very significant negative correlation with the dry matter content of the leaves. Therefore, we suspected that the reason why the correlation between specific leaf area and dry matter content first increases and then decreases may be the allocation strategy of plants to their limited resources when they were subject to changes in the external environment. We analyzed that under the moderate temperature (T1), plants with smaller leaf area used more resources to construct the defense structure in order to prevent high temperature and water loss, thus increasing the investment in the defense structure. This was reflected in the increase of dry matter content in the leaves [54, 55]. On the contrary, if the temperature was too high (T2), plants will use a small part of their resources for photosynthesis investment in order to maintain their survival, thus reducing the resources for building defense structures [40, 55].

## Conclusion

This field-based study, which evaluated the response of urban greening plant functional traits to climate warming and its adaptation strategies. Different from other researches, we took mature trees as the research object and carried out experiments in an open urban environment. Several findings are worth noting. Firstly, leaf functional traits exhibit relatively consistent trade-offs under a warming environment, they adapt to such an environment by adjusting their leaf traits and structures in limited resources. Secondly, the relationship between plant functional traits was basically consistent with the global scale, moderate warming could enhance the correlation between them to a certain extent, while excessive warming will weaken its correlation in the urban environment. Finally, this study illustrated the survival strategies adopted by plants to adapt to the high-temperature environment in the city. At the same time, we further verified the existence of the global leaf economics spectrum, which belongs to “ Quick investment-return type”. The adaptation strategy of urban plants to warming was to reduce specific leaf area, stomatal size and stomatal aperture, and increase chlorophyll content, leaf tissue density, leaf dry matter content and stomatal density. This will provide a theoretical basis for the allocation of urban plants and the management of tending in an environment of deepening urbanization.

## Methods

### Sites and sampling

**Fig. 5:**
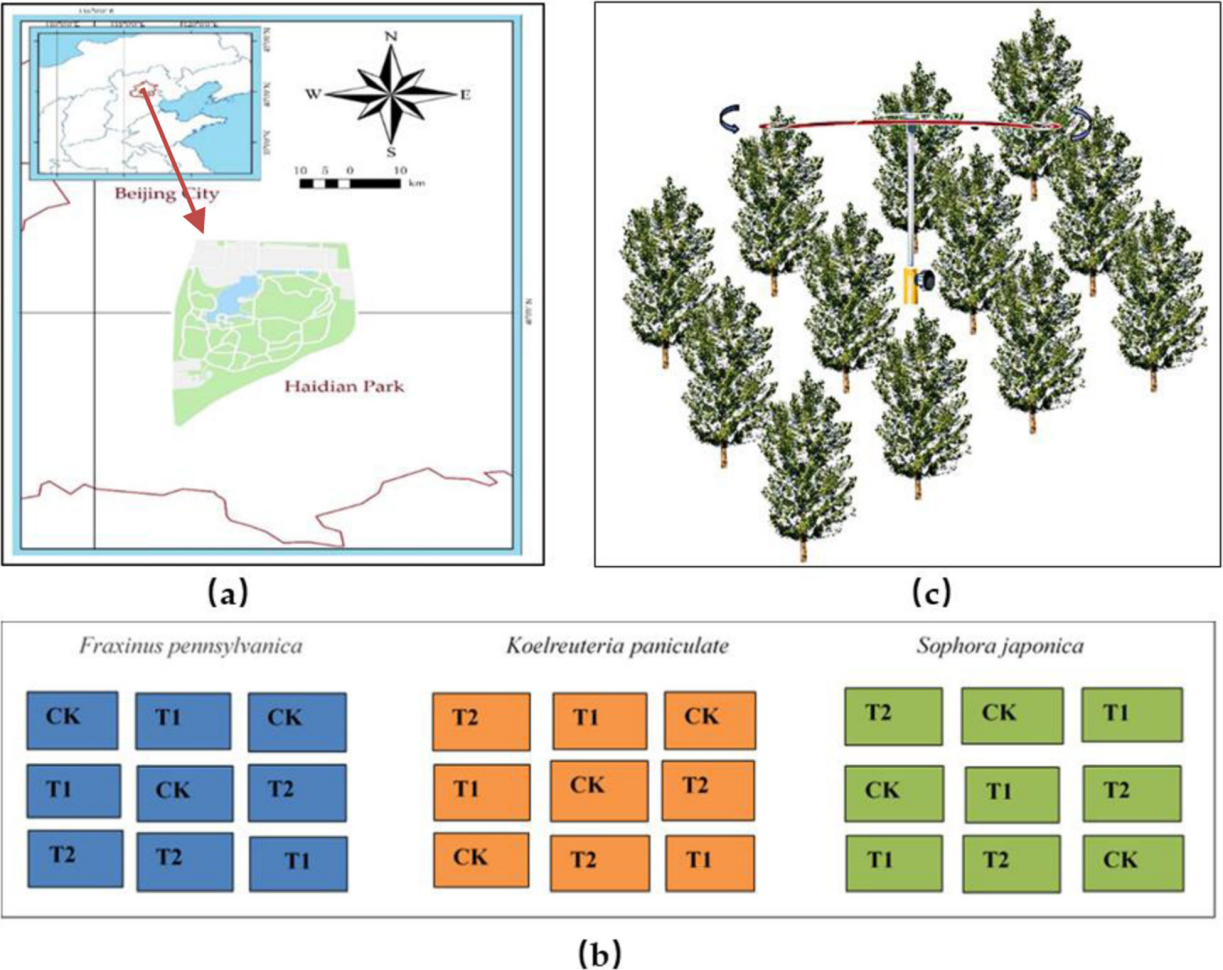
**a** Location map of the study site. The research site is located in Haidian Park (The map of Haidian Park is downloaded from 360 Map Natwork: https://ditu.so.com/?pid=10d2573a0e802bb5&src=onebox. The boundary of the area is automatically generated by 360 map website, and we have not made any changes.). **b** The experimental design diagrams. CK: Without warming; T1: Moderate warming; T2: Severe warming. **c** Schematic diagram of the warming device. The infrared heater is installed in the center of the woodland and is equipped with an automatic rotating device

Beijing, the capital of China, is located between longitudes 115°125′ and 117°130′E, and between latitudes 39°28′and 41°05′N. The climate is typical of the semi-humid continental monsoon climate in the northern temperate zone. The annual average temperature is 10~14 °C, the average summer temperature is 27.5 °C, and the annual average precipitation is about 610 mm (Data from China Meteorological Network: http://www.cma.gov.cn/). As shown in Fig. 5, the test site is located in Haidian Park (39°54′N, 116°25′E), Haidian District, Beijing, China, which meet the characteristics of the urban environment (Fig. 5(a)). The main species in the study area including *Fraxinus pennsylvanica, Koelreuteria paniculata, Sophora japonica,* and *Ailanthus altissima*. The test area was within 30km^2^, which ensures the relative consistency of the environment and daily management.

In mid-March 2018, a representative plot with uniform vegetation distribution was selected in the park, and 8~10 years old *Fraxinus pennsylvanica, Koelreuteria paniculata* and *Sophora japonica* (average height is 5 m) with good growth and uniform specifications were selected as test materials. Specific information is shown in Table 3 (Identification information refers to Flora of China).

**Table 3:** Identification information of the plant material used in your study

Tree species	Identification of the plant material
*Fraxinus pennsylvanica*	*Fraxinus pennsylvanica*, deciduous trees, 10–20 m high; Bark gray, rough, wrinkled. Branchlets reddish brown, cylindrical, yellow pilose or bald, old branches reddish-brown, smooth and glabrous. Feather compound leaves are 18-44 cm long. Petiole 2-5 cm long, base hardly inflated.
*Ailanthus altissima*	*Ailanthus altissima,* deciduous tree, with a height of more than 20 m, has smooth and straight bark. Leaves are odd-pinnate compound leaves, 40-60 cm long, petioles 7–13 cm long and lobules 13–27. Small leaves are opposite or nearly opposite, papery, ovate-lanceolate, 7–13 cm long, 2.5–4 cm wide, apex long acuminate, base oblique, truncated or slightly rounded, with 1 or 2 coarse serrations on each side, one gland on the back of the tooth, dark green on the leaf surface, grayish-green on the back, and stinking after soft crushing.
*Sophora japonica*	*Sophora japonica*, a tall tree, has the largest planting area in Beijing. Its bark is grayish-brown with cracks. The leaves are pinnately compound leaves with a length of 25 cm. Lobules are oval oblong in shape, opposite or alternate, 2.5-6 cm long and 1.5-3 cm wide. The back of the leaf is grayish-white with short hairs. 2 small stipules, drill-shaped. Panicle terminal is pyramid-shaped, up to 30 cm long.

### Test methods

#### Temperature increase test

In the experiment, we set up three gradients of control group (CK) and warming group (T1 and T2), and 30 replicates were set for each treatment. A total of 810 trees were tested (Fig. 5(b)). In March 2018, a temperature increasing device was set up in the test site, and a set of infrared radiant heaters was installed in the center of each sub-zone of the warming group (1000 W, 4 m, JFT220–1000, HS-2420, Kalglo Electronics, Bethlehem, USA). The warming effect was controlled according to the suspension height of the heater. The infrared radiant heater was installed at a distance of 1.5 m (T2) and 2 m (T1) from the top of the canopy, respectively. The effective warming zone of the warming device was approximately 5 m × 5 m. In order to avoid the interference of rain, the transparent lamp cover was installed above the lamp tube. The non-warming group installed an empty lampshade in the manner of a warming group in order to eliminate the difference between the warming treatments.

In the experimental area, we managed all the trees in a unified way. Apart from normal watering, no human interference was involved. Here, the management of watering was also consistent. Therefore, we control the temperature as the leading factor of the environment in order to further study the response of urban greening plants to warming. The temperature increase treatment of this test began in July 2018 and continued to increase until the end of October. During the test, all the seedlings were managed in the same way to ensure the relative consistency of water and nutrients. The suspension height of the heating instrument was appropriately adjusted according to the growth height of the plant and the change of the natural temperature. In addition, we installed an automatic rotating device at the junction of the lamp, and set a uniform speed (30 min/r) rotation to ensure uniform temperature increase of each test sample, which can avoid the temperature concentration directly under the infrared heater adversely affecting the growth of some plants (Fig. 5(c)).

#### Environmental factor monitoring

Environmental indicators were atmospheric temperature and soil temperature. The temperature and humidity recorders (DS1923G, Maxim/Dallas Semiconductor, Dallas, USA) were installed in various districts in late June 2018. Data were automatically recorded every hour. The recorder was installed at a distance of 50 cm from the ground and a transparent plastic baffle was installed 10 cm above it to prevent the effects of watering. A thermocouple (Heraeus, ST-1-PT1000, Germany) was placed 1 cm below the center of each plot to determine the soil temperature, and an automatic data collector (Heraeus, ST-1-PT1000, Germany) was set to record data every hour. Continuous monitoring at 24 h.

**Fig. 6:**
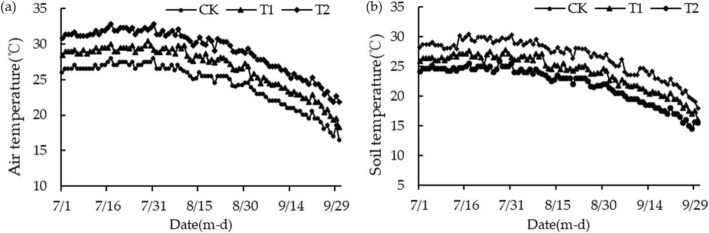
Heater-induced changes in daily mean soil moisture and daily mean soil temperature during the growing season. **a**: Daily mean soil moisture, **b**: Daily mean soil temperature

The data showed that the average temperature in Beijing in 2017 was 12.6 °C, which was 1.1 °C higher than the average of the year (1981–2010) and 0.5 °C higher than that in 2016 (Data were quoted from China Meteorological Network: http://www.cma.gov.cn/). It was the highest historical value in 2014, with more precipitation. The number of hours of sunshine was normal, and the number of days of high temperature was significantly higher. Compared with CK, the average temperature of T1 and T2 in the warming group increased by 2.5 °C (*P* < 0.01) and 5.6 °C (*P* < 0.01) during the whole growing season, respectively. The daily maximum temperature also increased by 2.7~3.5 °C and 3.9~5.8 °C respectively. Compared with CK, the soil temperature of T1 and T2 in the warming group increased by 2.4 °C (*P* < 0.01) and 4.6 °C (*P* < 0.01), respectively. This indicated that the warming effect of the warming group was highly significant (Fig. 6).

#### Determination of leaf functional traits

Based on the study of plant functional traits at global scale, we selected seven indicators that are sensitive to environmental changes and have greater plasticity: specific leaf area, chlorophyll content, leaf dry matter content, leaf tissue density and stomatal functional traits (stomatal density, stomatal area, and stomatal aperture) [17, 53, 54]. After the warming process, the leaves were collected in sunny weather from 07:00–08:30 in October 2018. A total 30 healthy and mature leaves were randomly selected from each tree. Temporary slides were made based on the temporary nail polish imprint method. First, moisture and dust attached to the surface of the leaves were removed by using a sanitary cotton ball, and then the transparent main nail oil was evenly applied to the leaf. After the imprinting liquid was dried in 3 to 5 min, the temporary film was made by tearing the imprinted film with pointed tweezers. Microscopic images of the stomata were taken using an optical microscope (BD-SW4001, Germany), and each slide was photographed with one field of view. A total of 900 stomatal microscopic images were collected in this experiment. Finally, the stomatal density (number·cm^− 2^), stomatal size (μm^2^), and stomatal aperture (μm) were further calculated according to the method of Zhu et al. [56].

Sixty healthy, fully-expanded leaves were randomly selected from each tree, placed them in a 5 °C icebox, and immediately brought them back to the laboratory to complete the determination of leaf functional traits within 1 h. The collected fresh leaves were measured for leaf thickness by upper, middle and lower portions using an electronic vernier caliper. Leaf thickness was measured by using the electronic vernier caliper. The petioles were cut in deionized water and placed in a dark environment at 5 °C for 12 h, then the moisture and impurities of the leaves were dried with absorbent cotton, and the leaf saturated fresh weight was weighed using an electronic balance. Leaf area was scanned using a leaf area scanner (Microtek, phantom k8, China) and the leaf area was calculated using Leaf area image analysis software. The leaf volume was calculated using the drainage method. The chlorophyll content was determined by an acetone-ethanol 1:1 extraction method using an atomic spectrophotometer. Finally, all the leaves were placed in a 105 °C oven and then transferred to 80 °C to dry to constant weight, and the leaf dry mass was weighed. Leaf dry matter content = Leaf dry weight/ Leaf saturated fresh weight, g·g^− 1^. Leaf tissue density = Leaf dry mass / Leaf volume, g·cm^− 3^. Specific leaf area = Leaf area / Leaf dry quality, cm^2^·g^− 1^. All leaf functional traits were tested in the laboratory of Beijing Forestry University.

#### Data processing

One-way ANOVA was used to determine the correlation between plant functional traits among different environmental factors. Pearson correlation coefficient was used to test the correlation between plant functional traits. Data analysis of variance, correlation analysis, significance analysis, and mapping were performed using SPSS 20.5 Software and EXCEL 2016 software, and using PowerPoint 2019 software for picture compositing and typesetting.

## Data Availability

The raw/processed data required to reproduce these findings cannot be shared at this time as the data also forms part of an ongoing study, but there are available from the corresponding author on reasonable request.
